# Exposure to residential greenness and subsequent risk of common mental disorders among individuals with COVID-19 in South Korea: A nationwide cohort study

**DOI:** 10.1371/journal.pone.0359016

**Published:** 2026-10-06

**Authors:** Dongjin Yeo, Selin Woo, Juyeong Kim, Kyeongeun Kim, André Hajek, Lee Smith, Sooji Lee, Seohyun Hong, Ho Geol Woo, Yerin Hwang, Dong Keon Yon

**Affiliations:** 1 Department of Medicine, Kyung Hee University College of Medicine, Seoul, South Korea; 2 Center for Digital Health, Medical Science Research Institute, Kyung Hee University College of Medicine, Seoul, South Korea; 3 Department of Regulatory Science, Kyung Hee University, Seoul, South Korea; 4 Department of Precision Medicine, Kyung Hee University College of Medicine, Seoul, South Korea; 5 Department of Health Economics and Health Services Research, University Medical Center Hamburg-Eppendorf, Hamburg Center for Health Economics, Hamburg, Germany; 6 Centre for Health, Performance and Wellbeing, Anglia Ruskin University, Cambridge, United Kingdom; 7 Department of Public Health, Faculty of Medicine, Biruni University, Istanbul, Turkey; 8 Department of Neurology, Kyung Hee University Medical Center, Kyung Hee University College of Medicine, Seoul, South Korea; 9 Department of Pediatrics, Kyung Hee University College of Medicine, Seoul, South Korea; University of Turin: Universita degli Studi di Torino, ITALY

## Abstract

**Background:**

Residential greenness is considered protective for mental health, but its impact on clinically diagnosed psychiatric disorders following COVID-19 remains unclear. This study investigated the association between residential greenness and risk of common mental disorders after COVID-19, and whether this relationship differed by population subgroup.

**Methods:**

We conducted a nationwide cohort study of 3,097,179 adults aged ≥19 years with confirmed COVID-19 in South Korea between 2018 and 2023 using the K-COV-N cohort, which integrates administrative and clinical data from the National Health Insurance Service and COVID-19 data from the Korea Disease Control and Prevention Agency. Residential greenness was assessed using the normalized difference vegetation index (NDVI) and categorized as low (0.2– < 0.4), moderate (0.4– < 0.6), or high (≥0.6). The primary outcome was first diagnosis of anxiety disorder, depression, insomnia, or bipolar disorder ≥30 days post-infection. Weighted Cox proportional hazards models with inverse probability of treatment weighting were used to estimate adjusted hazard ratios (aHRs).

**Results:**

Compared to low NDVI, moderate NDVI was associated with reduced risk of any common mental disorder (aHR, 0.97; 95% CI, 0.96–0.98), with the strongest effect for bipolar disorder (0.84; 0.79–0.88). High NDVI showed no overall benefit (1.00; 0.99–1.01) and was associated with increased anxiety risk (1.04; 1.03–1.06), especially in older adults and those with comorbidities. Younger individuals experienced the greatest benefit from moderate greenness.

**Conclusion:**

Moderate residential greenness was linked to lower risk of mental disorders following COVID-19, while high greenness conferred limited benefit and potential risk. Greening strategies should be paired with strengthened health and social infrastructure to reduce disparities.

## Introduction

Studies have consistently shown that individuals diagnosed with COVID-19 are at an increased risk of developing mental disorders, including anxiety, depression, sleep disorders, and cognitive decline.[1,2] While these outcomes may be related to direct biological effects of the virus, emerging evidence also suggests that aspects of the residential environment, such as green space, may have influenced mental health outcomes during the pandemic.[3,4] Public health measures, including lockdowns and mobility restrictions, confined individuals to their residential surroundings for extended periods, amplifying the potential influence of local environmental features on mental health.[2,4,5]

Greenness may influence mental health through biological, environmental, and behavioral pathways, including stress reduction, improved environmental conditions, and increased physical activity and social interaction, as described in previous studies.[2,3] However, only a limited number of studies have examined whether residential greenness is associated with clinically diagnosed mental disorders following SARS-CoV-2 infection.[1,2,6] These studies have used different approaches, including mobility-based measures of use, satellite-derived indices of surrounding vegetation, and self-reported nature experiences, [1,2,6] representing different aspects of environmental exposure and reflecting behavioral and environmental pathways.[7] However, they have relied on self-reported outcomes or have not examined a broad range of mental disorders. Moreover, it remains unclear whether these associations differ across population subgroups, including age, sex, or the presence of comorbid conditions.[1,2,6]

To address this gap, we conducted a nationwide, population-based cohort study in South Korea using linked administrative and clinical data from the National Health Insurance Service (NHIS) of South Korea, COVID-19-related data from the Korea Disease Control and Prevention Agency (KDCA), and residential greenness data from the satellite imagery based on GPS coordinates. We investigated whether residential greenness, as measured by NDVI, was associated with the risk of newly diagnosed common mental disorders, including anxiety, depression, insomnia, and bipolar disorder, among individuals with COVID-19. By integrating objective environmental exposure data with large-scale clinical records and conducting stratified analyses by age and comorbidity burden, this study provides novel insights into the potential mental health implications of residential greenness in the post-COVID-19 era.

## Methods

### Data source

We linked the K-COV-N cohort and residential greenness data from the satellite imagery based on GPS coordinates. The K-COV-N cohort was developed through a collaboration between the NHIS of Korea and KDCA. It combined national health screening data and healthcare utilization records from the NHIS with COVID-19-related data from the KDCA, covering the period from Jan 1, 2018, to Dec 31, 2023. South Korea’s universal single-payer health system enabled near-complete population coverage, allowing the K-COV-N cohort to support longitudinal follow-up across healthcare settings and to capture a wide range of clinical events, including those occurring outside medical institutions.

### Study population

This study used data from the K-COV-N cohort covering the period from 2018 to 2023. We identified individuals aged 19 years and older with confirmed SARS-CoV-2 infection (Fig 1(A)). The index date was defined as the date of confirmed SARS-CoV-2 infection. Because NDVI exposure was assigned based on the residential district, only individuals whose residential district was unchanged between the calendar year preceding the index date and the calendar year of the index date were included to minimize exposure misclassification due to residential mobility. Pre-existing conditions were identified using data from Jan 1, 2018, up to the index date. Individuals were excluded if they had a prior diagnosis of the corresponding outcome before the index date or had missing data on key covariates. Follow-up for the primary outcome began 30 days after the index date and continued until the earliest of the following: (1) death, (2) the first occurrence of the outcome of interest, or (3) Dec 31, 2023 (Fig 1(B)). This study was approved by the Institutional Review Board of Kyung Hee University (KHSIRB-23–241[EA]) and was conducted under the authorization of the KDCA and NHIS (KDCA-NHIS-2024-1-280). All analyses were conducted using de-identified administrative data, and the requirement for informed consent was waived in accordance with applicable regulations. The data for this study were accessed on June 09, 2025, for research purpose. The research team had no access to individually identifiable participant data during or after collection, thereby ensuring strict confidentiality and adherence to ethical standards.

**Fig 1:**
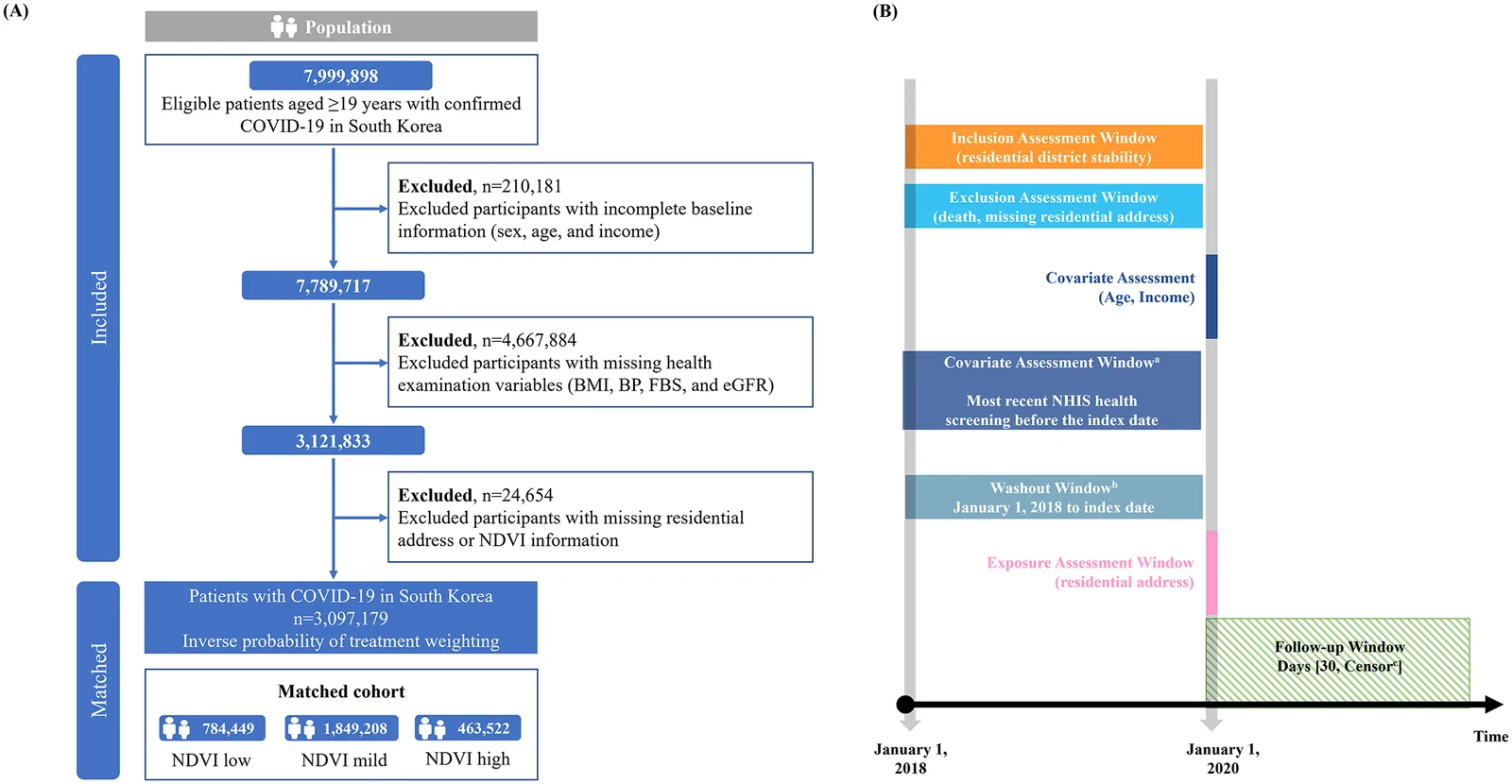
(A) Flow chart and (B) study diagram. Abbreviations: BMI, body mass index; eGFR, estimated glomerular filtration rate; FBS, fasting blood glucose; NDVI, normalized difference vegetation index; NHIS, National Health Insurance Service. ^a^ Clinical measurements and lifestyle-related variables: body mass index, blood pressure, fasting blood glucose, estimated glomerular filtration rate, smoking status, alcohol consumption, and aerobic physical activity. ^b^ Washout period applied to identify incident cases by excluding individuals with a diagnosis of the corresponding outcome between January 1, 2018, and the index date. ^c^ Follow-up began 30 days after the index date and continued until the earliest occurrence of death, the first diagnosis of the outcome, or December 31, 2023.

### Exposure

Green space exposure was quantified using the NDVI, a satellite-derived measure of vegetation density. NDVI was selected because it provides an objective and spatially consistent measure of vegetation density at a nationwide scale, allowing standardized exposure assessment across regions. [8] As a measure of surrounding vegetation, NDVI is linked to environmental exposure pathways, including air quality, temperature, and noise conditions. [8] NDVI was calculated as the difference between near-infrared and visible radiation divided by their sum, reflecting the extent of photosynthetically active plant cover.[9] NDVI values were obtained from the Moderate Resolution Imaging Spectroradiometer (MODIS) product MOD13A3 (version 6.1), developed by the National Aeronautics and Space Administration’s Terra satellite. This dataset provides NDVI measurements at a 1-kilometer spatial resolution, available from January 2007 to December 2024. For this study, monthly NDVI values were calculated for all 282 administrative districts in South Korea (Si-Gun-Gu), which serve as functional units of local governance.[10] NDVI values were then assigned to individuals based on their residential administrative district. In this study, NDVI values ranged from 0.235 to 0.723 (mean, 0.48; standard deviation, 0.10). Consistent with previous epidemiological studies, [11,12] NDVI was categorized into three groups to enable comparison across different levels of residential greenness: 0.2 to 0.4 as the reference, 0.4 to 0.6 as moderate NDVI, and 0.6 or higher as high NDVI.

### Outcome

The primary outcome was defined as the first recorded diagnosis of a common mental disorder, identified using the International Classification of Diseases, 10th Revision (ICD-10). To ensure incident case ascertainment, individuals with a diagnosis of the corresponding outcome before the index date were excluded. To minimize reverse causation, only diagnoses occurring 30 days or more after confirmed SARS-CoV-2 infection were considered outcomes. Common mental disorders included anxiety disorders, insomnia, bipolar disorder, and depression. ICD-10 codes used to define each outcome were F40-F48 for anxiety disorders, F510 and G470 for insomnia, F30 and F31 for bipolar disorder, and F32, F33, and F341 for depression.

### Covariates

A comprehensive set of demographics, clinical, and behavioral covariates were included to adjust for potential confounding. Demographic information was obtained from the national health insurance database and comprised sex, age group (categorized as 19–39, 40–59, and ≥60 years), region of residence (urban and rural), and household income level, classified into tertiles (low [0–39th percentile], middle [40–79th percentile], and high [80–100th percentile]). Clinical measurements and lifestyle-related variables were obtained from the most recent NHIS health screening examination conducted before the index date. Laboratory values were derived from fasting blood samples collected during the examination. Body mass index (BMI) was categorized according to Asia-Pacific regional criteria: underweight (<18.5 kg/m²), normal weight (18.5–22.9 kg/m²), overweight (23.0–24.9 kg/m²), and obese (≥25.0 kg/m²).[13,14] Blood pressure was classified as normal (systolic <140 mmHg and diastolic <90 mmHg) or elevated (systolic ≥140 mmHg and/or diastolic ≥90 mmHg). Estimated glomerular filtration rate (eGFR) was categorized as <60 or ≥60 mL/min/1.73 m². Fasting blood glucose levels were divided into <100 or ≥100 mg/dL.[13,14] Health status indicators included the Charlson comorbidity index (CCI), use of antihypertensive medications, and treatment history for diabetes and dyslipidemia.[15] Lifestyle-related variables included smoking status (never, former/current smoker, or unknown), weekly alcohol consumption frequency (<1 day, 1–2 days, 3–4 days, ≥ 5 days, and unknown), and aerobic physical activity level. Physical activity was assessed using the Korean version of the modified Global Physical Activity Questionnaire (GPAQ), implemented as part of the National Health Insurance Service health screening program. [16] Participants reported the frequency and duration of moderate- and vigorous-intensity activities, which were used to calculate total metabolic equivalent (MET) minutes per week. [16] Sufficient physical activity was defined as ≥600 metabolic equivalent minutes per week, in accordance with WHO guidelines.[17] Missing values for smoking status, alcohol consumption, and physical activity were handled using the missing indicator method by creating an “unknown” category, whereas participants with missing values for all other covariates were excluded from the analysis.[18]

### Inverse probability of treatment weighting

To ensure comparability across NDVI categories, we applied inverse probability of treatment weighting (IPTW) using a 1:1:1 approach. Covariates included in the weighting model were age, sex, region of residence, income level, medication history, CCI, BMI, blood pressure, fasting blood glucose, eGFR, smoking status, alcohol consumption, and aerobic physical activity. Covariate balance after weighting was assessed using standardized mean differences (SMDs), with values less than 0.1 indicating acceptable balance between groups. Cohort characteristics before weighting are shown in **S1 Table in S1 File**, and IPTW-adjusted baseline characteristics across NDVI groups are presented in **Table 1**.

**Table 1:** 1:1:1 IPTW propensity score weighted characteristics of cohort (n=3,097,179).

Baseline characteristic	Total	T1^a^	T2^a^	SMD^b^ (T2 *vs.* T1)	T3^a^	SMD^b^ (T3 *vs.* T1)
Total	3,097,179	784,449	1,849,208		463,522	
Age (y), mean ± SD	51.21±13.16	51.18±26.00	51.22±16.97	0.002	50.66±34.95	0.017
Sex, n (%)				0.002		0.023
Men	1,900,439 (61.36)	-61.26	-61.35		-62.36	
Women	1,196,740 (38.64)	-38.74	-38.65		-37.64	
Region of residence, n (%)				0.001		0.017
Urban	1,386,228 (44.76)	-44.68	-44.75		-45.51	
Rural	1,710,951 (55.24)	-55.32	-55.25		-54.49	
Baseline income, n (%)				0.026		0.045
Q1	862,365 (27.84)	-28.58	-27.83		-26.56	
Q2	1,267,357 (40.92)	-40.56	-41.06		-41.94	
Q3	967,457 (31.24)	-30.86	-31.11		-31.50	
BMI, n (%)				<0.001		<0.001
Underweight	67,605 (2.18)	-2.22	-2.16		-2.16	
Normal	919,534 (29.69)	-29.55	-29.7		-29.51	
Overweight	752,944 (24.31)	-24.25	-24.32		-24.37	
Obesity	1,357,096 (43.82)	-43.99	-43.81		-43.96	
Charlson comorbidity index, n (%)			0.071		0.071	
0	2,838,086 (91.63)	-91.78	-91.63		-91.62	
1	144,143 (4.56)	-4.23	-4.66		-5.04	
≥2	114,950 (3.71)	-3.99	-3.71		-3.34	
Blood pressure, n (%)				0.011		0.020
SBP<140 and DBP<90	2,666,452 (86.09)	-86.46	-86.10		-87.15	
SBP≥140 or DBP≥90	430,727 (13.91)	-13.54	-13.90		-12.85	
Fasting blood sugar, n (%)				0.005		0.015
<100 mg/dL	1,778,195 (57.41)	-57.13	-57.40		-57.85	
≥100 mg/dL	1,318,984 (42.59)	-42.87	-42.60		-42.15	
Glomerular filtration rate, n (%)			0.003		0.009	
<60mL/min/1.73m²	168,011 (5.42)	-5.36	-5.43		-5.16	
≥60mL/min/1.73m²	2,929,168 (94.58)	-94.64	-94.57		-94.84	
Smoking status, n (%)				<0.001		<0.001
Never	3,069,767 (99.11)	-99.05	-99.13		-99.20	
Former/Current	26,096 (0.84)	-0.90	-0.83		-0.76	
Unknown	1,316 (0.04)	-0.05	-0.04		-0.04	
Alcohol consumption, n (%)				0.023		0.023
<1 day/week	1,895,444 (61.20)	-60.54	-61.51		-61.76	
1-2 day/week	845,226 (27.29)	-27.85	-27.16		-26.94	
3-4 day/week	274,278 (8.86)	-8.97	-8.72		-8.64	
≥5 day/week	80,862 (2.61)	-2.58	-2.56		-2.60	
Unknown	1,369 (0.04)	-0.05	-0.04		-0.05	
Physical activity, n (%)				0.020		0.060
Insufficient	1,393,740 (45.00)	-45.62	-45.15		-99.20	
Sufficient	1,702,131 (54.96)	-54.33	-54.81		-0.76	
Unknown	1,308 (0.04)	-0.05	-0.04		-0.04	
Medication for hypertension, n (%)			0.002		0.018	
No	2,453,524 (79.22)	-79.27	-79.21		-79.98	
Yes	643,655 (20.78)	-20.73	-20.79		-20.02	
Medication for diabetes mellitus, n (%)			0.001		0.012	
No	2,834,723 (91.53)	-91.50	-91.52		-91.83	
Yes	262,456 (8.47)	-8.50	-8.48		-8.17	
Medication for dyslipidemia, n (%)			0.002		0.007	
No	2,769,705 (89.43)	-89.49	-89.42		-89.72	
Yes	327,474 (10.57)	-10.51	-10.58		-10.28	

Abbreviations: BMI, body mass index; NDVI, normalized difference vegetation index; SD, standard deviation; SMD, standardized mean difference.

^a^ T1, low NDVI group; T2, medium NDVI group; T3, high NDVI group.

^b^ SMD < 0.1 indicates no major imbalance.

### Statistical analysis

We examined the association between residential greenness, categorized by NDVI, and the incidence of common mental disorders among individuals with confirmed COVID-19. The index date was defined as the date of SARS-CoV-2 diagnosis, and follow-up continued until the earliest of outcome occurrence, death, or Dec 31, 2023. To address potential confounding across NDVI categories, IPTW based on propensity scores was applied. Covariates included in the propensity score model were age, sex, region of residence, household income, medication history, CCI, BMI, BP, FBG, GFR, smoking status, alcohol consumption, and aerobic physical activity. Weighted Cox proportional hazards regression models were used to estimate adjusted hazard ratios (aHRs) and corresponding 95% confidence intervals (CIs) for each mental disorder outcome across NDVI categories. Models were further adjusted for the same covariates included in the propensity score model. To formally assess dose-response relationships, tests for linear trend across NDVI categories were additionally performed. Interaction terms were also included to evaluate effect modification across prespecified stratification variables, and corresponding p-values for interaction were calculated. Statistical significance was defined as a two-sided p-value of less than 0.05. All analyses were conducted using SAS software (version 9.4; SAS Institute Inc., Cary, NC, USA).

## Results

### Study population

A total of 7,999,898 adults aged 19 years and older with confirmed COVID-19 in South Korea were assessed for eligibility. A total of 4,902,719 individuals were excluded due to missing residential address data, death prior to the index date, or inconsistent address records in the year preceding infection. After exclusions, 3,097,179 eligible individuals were classified into exposure groups according to levels of residential NDVI. To ensure comparability across NDVI categories, 1:1:1 IPTW was applied, resulting in a final matched cohort of 361,044 individuals in each of the low, moderate, and high NDVI groups.

### NDVI exposure and risk of common mental disorders

Moderate levels of residential NDVI were associated with a lower risk of common mental disorders (aHR, 0.97 [95% CI, 0.96–0.98]), corresponding to an absolute difference of approximately 1.04 fewer cases per 1,000 person-years compared with the low-NDVI group, while high NDVI was not associated with overall risk (aHR, 1.00 [95% CI, 0.99–1.01]). Associations differed across specific mental outcomes. For insomnia, both moderate (aHR, 0.97, [95% CI, 0.95–0.98]) and high NDVI groups aHR, (0.97 [95% CI, 0.95–0.98]) were associated with lower risk. The strongest inverse association was observed for bipolar disorder, with aHRs of 0.84 (95% CI, 0.79–0.88) and 0.78 (95% CI, 0.74–0.82) in the moderate and high NDVI groups, respectively. Similar patterns were observed for depression. In contrast, for anxiety disorders, moderate NDVI was associated with reduced risk (aHR, 0.98 [95% CI, 0.96–0.99]), while high NDVI was associated with increased risk (aHR,1.04 [95% CI, 1.03–1.06]). Detailed effect estimates for each outcome are presented in Figs 2 and 3 and S2 Table in S1 File.

**Fig 2:**
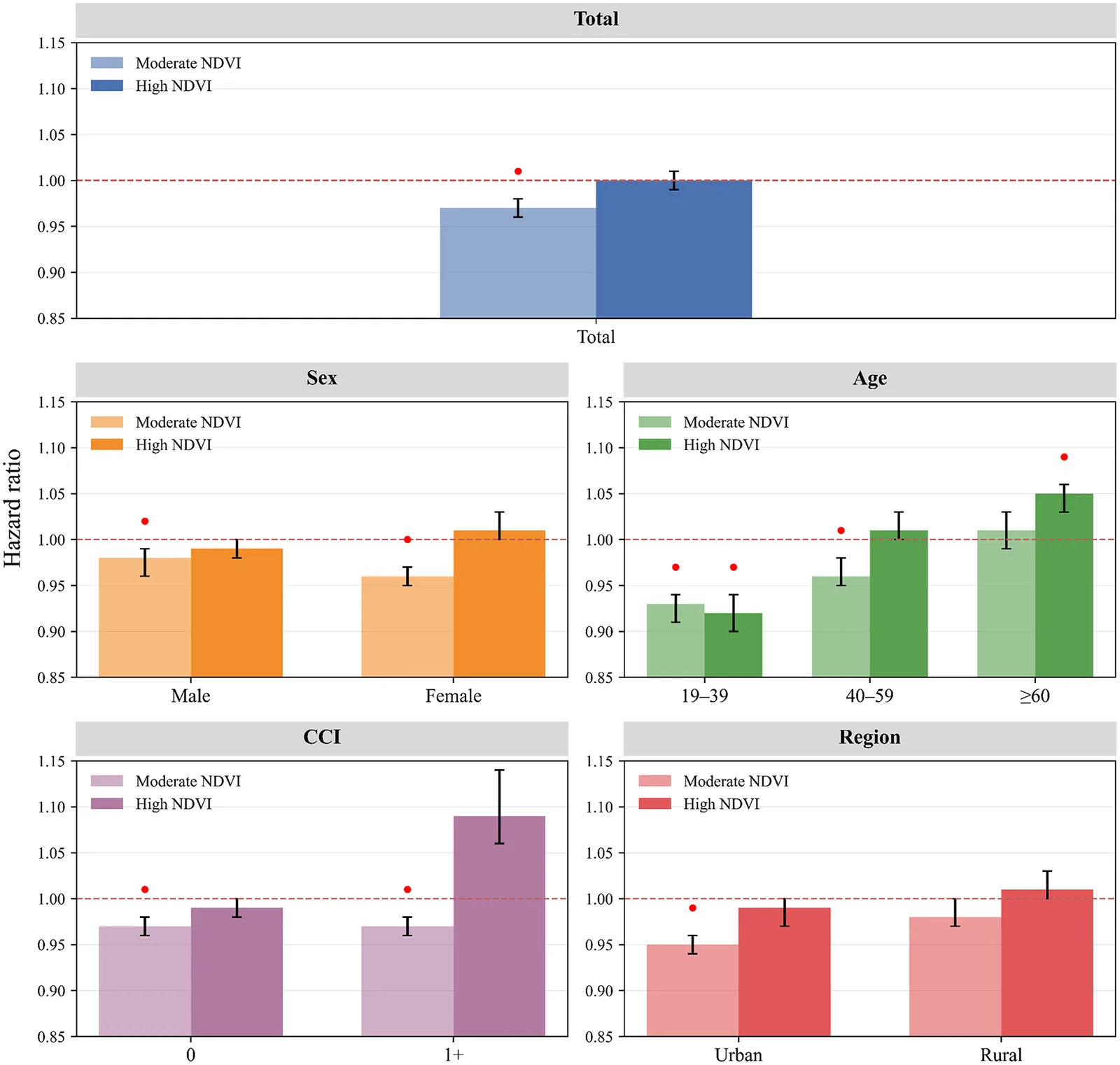
Hazard ratios for common mental disorders by NDVI exposure stratified by sex, age, comorbidity, and region burden. Abbreviations: CCI, Charlson comorbidity index; NDVI, normalized difference vegetation index.

**Fig 3:**
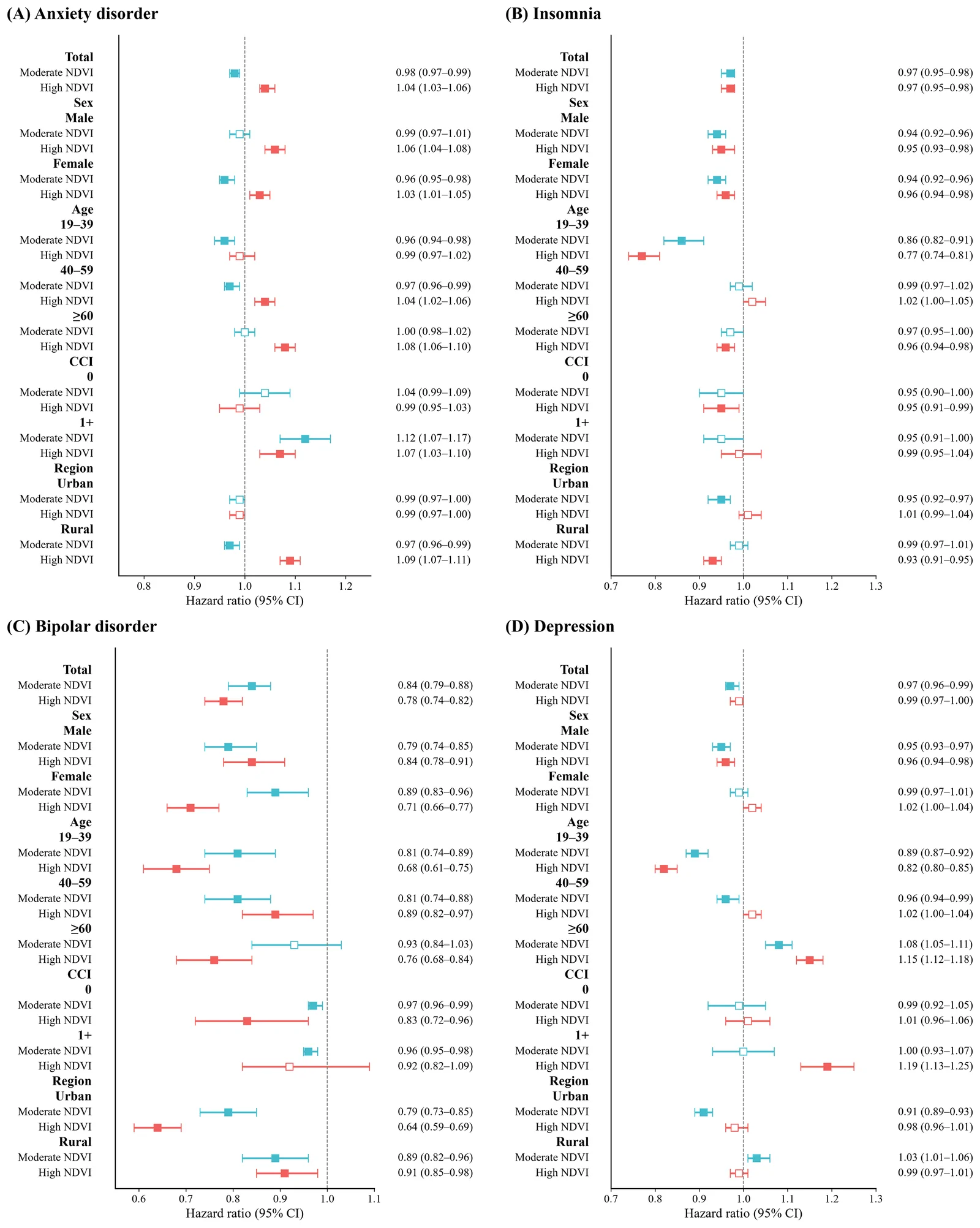
Hazard ratios for (A) anxiety disorder, (B) insomnia, (C) bipolar disorder, and (D) depression by NDVI exposure and covariates. Filled markers indicate statistically significant associations. Abbreviations: CCI, Charlson comorbidity index; CI, confidence interval; NDVI, normalized difference vegetation index.

### Stratification analysis

Associations between residential NDVI and common mental disorder risk varied by sex, age, and comorbidity burden. Overall common mental disorder risk estimates were similar for males and females. Among younger adults aged 19–39 years, moderate NDVI was associated with the lowest risk across most outcomes, including insomnia, bipolar disorder, and depression, although the associations became less pronounced with increasing age. For example, the aHR for common mental disorders at moderate NDVI increased from 0.93 (95% CI, 0.91–0.94) in younger adults to 0.96 (95% CI, 0.95–0.98) in those aged 40–59 years, while high NDVI was associated with increased risk in adults aged 60 years and older (aHR, 1.05 [95% CI, 1.03–1.06]). This age gradient was most pronounced for anxiety disorder and depression. Similar trends were observed according to comorbidity status: while moderate NDVI was associated with reduced risk regardless of comorbidity, high NDVI was linked with elevated mental disorder risk among individuals with pre-existing conditions (aHR, 1.09 [95% CI, 1.06–1.24]), compared to a null association among those without (aHR, 0.98 [95% CI 0.98–1.00 In region-stratified analyses, moderate NDVI was associated with a lower risk of common mental disorders among both urban (aHR, 0.95 [95% CI, 0.94–0.96]) and rural (aHR, 0.98 [95% CI, 0.97–1.00]) residents. Detailed effect estimates for each outcome are exhibited in Figs 2 and 3 and S3-S6 Tables in S1 File.

## Discussion

### Key findings

This is the first study to evaluate associations between residential greenness and the development of common mental disorders among individuals diagnosed with COVID-19, using a nationwide cohort of over 2.3 million individuals in South Korea. First, we observed a non-linear association between residential NDVI and common mental disorder risk. Moderate levels of NDVI were associated with lower risk, whereas high levels showed no significant association with overall mental health burden. Second, in contrast, associations at high NDVI levels differed across individual outcomes, with higher risk of anxiety disorders, lower risk for insomnia and bipolar disorder, and no significant association for depression. Third, among specific outcomes, bipolar disorder showed the strongest inverse association with moderate NDVI, particularly among individuals younger than 60 years of age and those with comorbidities. Fourth, insomnia also showed lower risk with increasing NDVI, with the strongest inverse association observed among individuals aged between 19 and 39 years. These findings indicate a non-linear association between greenness and mental health, with differences across mental disorders and population subgroups.

### Comparisons with previous studies

Previous studies have reported inconsistent associations between residential greenness and mental health. Some found protective effects, while others reported no association or even adverse outcomes.[4,19,20] These discrepancies may reflect differences in study design, population characteristics, and how greenness exposure and mental health were defined.[19] While several cross-sectional studies conducted during the COVID-19 pandemic found lower psychological distress among those living in greener areas, many relied on self-reported measures of mental health, which limit diagnostic specificity and are susceptible to reporting bias.[1,6,21] One cohort study used clinical diagnoses but defined greenness by proximity to green space rather than vegetation density, and mental outcomes were not assessed separately.[2] By contrast, our study used satellite-derived NDVI to quantify residential greenness and examined clinically diagnosed mental disorders individually.[22,23] This approach enabled more precise exposure assessment and outcome classification, and may help explain prior inconsistent findings.

We observed a non-linear association, with moderate, but not high, levels of greenness associated with lower mental disorder risk. At higher NDVI levels, associations differed across outcomes, with a small increase observed for anxiety disorders. This finding aligns with differences in what NDVI represents across settings, as higher values indicate vegetation density without indicating accessibility or use. In South Korea, higher NDVI values are more likely to correspond to rural contexts, where healthcare access and utilization differ and environmental exposures also vary, contributing to the observed associations.[24–26]

### Plausible underlying mechanisms

Residential greenness may influence mental health through a range of interconnected biological and environmental pathways. Exposure to vegetated environments has been associated with reduced psychological stress, including lower cortisol levels and improved emotional regulation among individuals residing in greener areas.[27] Greenness may also modify exposure to environmental stressors, particularly air pollution. Vegetated areas are generally associated with lower concentrations of fine particulate matter, which has been linked to increased risk of psychiatric disorders, including bipolar disorder, depression, anxiety, and insomnia.[28–31] These associations are though to involve systemic inflammation and oxidative stress, with exposure to green environments associated with downregulation of pro-inflammatory pathways, including nuclear factor kappa B, interleukin-6, and interleukin-1β.[2,28,32] In addition, greenness may influence mental health through behavioral pathways, where access to and interaction with green environments have been associated with higher levels of physical activity and lower risk of depression and anxiety and improved psychological well-being.[7]

However, the extent to which greenness confers mental health benefits likely depends on broader social and structural conditions. The COVID-19 pandemic imposed widespread psychosocial stressors, including prolonged social isolation and mobility restrictions, both of which can disrupt neuroendocrine function and activate inflammatory responses.[33] In such contexts, particularly in rural or high-greenness areas with limited access to mental health services and social support, the potential psychological benefits of greenness may be attenuated.[34,35] These findings underscore the importance of considering structural determinants when evaluating the mental health impacts of natural environments.

### Clinical and policy implications

Although the observed associations were modest in magnitude, our findings have implications for current greening policies in South Korea. National initiatives, including the Act on Urban Parks and Green Areas and the Creation and Management of Urban Forests Act, have focused on expanding neighborhood green spaces and urban forests to improve environmental quality and public health.[36,37] However, our findings suggest that increasing vegetation density may not necessarily result in additional mental health benefits, as moderate levels of residential greenness were associated with the lowest risk of common mental disorders, whereas higher NDVI was not associated with further reductions in overall risk. This distinction may be particularly relevant in South Korea, where areas with the highest NDVI often correspond to mountainous or rural regions rather than accessible neighborhood green spaces and may have more limited access to mental health services and community resources than metropolitan areas.[24–26] Disparities such as these were likely compounded during the COVID-19 pandemic, when mobility restrictions further constrained access to both services and social supports. Therefore, as urban greening initiatives expand, complementary efforts are needed to strengthen local healthcare and social infrastructure, especially in regions where these resources are limited.[38,39] In addition, features of high-quality green environments, including accessibility, safety, and usability, are likely important for mental health benefits associated with greenness, given that higher NDVI may not correspond to accessible or usable green space.[40]

### Strengths and limitations

This nationwide cohort study of 2,296,641 adults with confirmed COVID-19 in South Korea examined the association between residential NDVI and subsequent mental health outcomes, using comprehensive administrative and satellite-derived data. However, several limitations should be considered when interpreting the findings. First, common mental disorder diagnoses were identified using ICD-10 diagnostic codes from national health insurance claims data. [41,42] Because the database relies exclusively on ICD-10-coded claims, diagnoses that were not recorded using ICD-10 codes could not be ascertained. In addition, ascertainment of pre-existing mental disorders was limited by the availability of the K-COV-N database, which contains records from Jan 1, 2018, onward. Consequently, some participants with mental disorders diagnosed before this period may have been misclassified as incident cases. Nevertheless, to minimize this potential bias, we excluded individuals with a diagnosis of the corresponding outcome before the index date and considered only the first diagnosis recorded at least 30 days after confirmed SARS-CoV-2 infection as an incident outcome. Second, greenness exposure was assessed using NDVI at the administrative district (Si-Gun-Gu) level, which may not represent individual residential exposure, particularly given variation in district size and differences in greenness within districts. In addition, the 1-kilometer spatial resolution of the MODIS data may not describe fine-scale variation in residential surroundings. Although within-district variation could not be accounted for, exposure was assigned based on residential administrative districts across the nationwide cohort, allowing consistent assessment of greenness across all regions.[43] Third, NDVI exposure was assigned based on residential address, assuming individuals spent most of their time at home. Although this may not apply to all participants, particularly those who work or spend substantial time away from home, mobility restrictions during the pandemic likely reduced daily variability in exposure, making this a reasonable approximation for environmental context. Fourth, we did not include a comparison group of individuals without COVID-19, as the study was designed to evaluate associations with mental health outcomes within individuals with confirmed SARS-CoV-2 infection. As a result, it is not possible to determine whether the observed associations between greenness and psychiatric outcomes are specific to the post-COVID-19 context or represent broader environmental influences on mental health. [44,45] Fifth, although we adjusted for a comprehensive set of demographic, clinical, and behavioral covariates, residual confounding cannot be excluded. In particular, environmental factors such as particulate matter concentration, as well as contextual variables including noise exposure or access to healthcare services were not available in the dataset and may influence the observed associations between greenness and mental health outcomes. [8,28]

Despite these limitations, to our knowledge, this is among the first nationwide studies to examine the relationship between local environmental conditions and mental health outcomes after COVID-19, with implications for future public health and urban planning.

## Conclusion

This nationwide study found that moderate levels of residential NDVI were associated with a reduced risk of common mental disorders among adults with COVID-19. In contrast, high levels of NDVI were not consistently associated with better mental health and were linked to increased risk in vulnerable groups, including older adults and those with underlying health conditions. These findings indicate that the mental health impacts of greenness vary across populations and contexts. Urban greening strategies should be implemented alongside efforts to improve healthcare access and strengthen social support systems to promote mental health more effectively and equitably.

## Supporting information

S1 File Supporting information. Contains S1–S6 Tables.
(DOCX)
