# Supplementary material for: Exposure to residential greenness and subsequent risk of common mental disorders among individuals with COVID-19 in South Korea: A nationwide cohort study
Source: PLoS One. 2026 Oct 6;21(10):e0359016. doi: 10.1371/journal.pone.0359016 (PMC13641331; doi:10.1371/journal.pone.0359016)
Supplement: Supplementary file 1 [file pone.0359016.s001.docx]

**Supplementary Material**

Original Article

**Exposure to residential greenness and subsequent risk of common mental disorders among individuals with COVID-19 in South Korea: A nationwide cohort study**

**Running title:** NDVI exposure and common mental disorders in individuals with COVID-19

Dongjin Yeo^1,2&^, Selin Woo^1,2&^, Juyeong Kim^2,3^, Kyeongeun Kim^2,4^, André Hajek^5^, Lee Smith^6,7^, Sooji Lee^1,2^, Seohyun Hong^1,2^, Ho Geol Woo^7^, Yerin Hwang^2*^, Dong Keon Yon^1,2,3,4,8*^

^&^ These authors contributed equally.

^*^ These authors jointly supervised this work.

***Corresponding author**

**Dong Keon Yon,** MD, PhD, FACAAI, FAAAAI, ATSF (lead contact)

Department of Pediatrics, Kyung Hee University College of Medicine, 23 Kyungheedae-ro, Dongdaemun-gu, Seoul 02447, South Korea

Email: [yonkkang@gmail.com](mailto:yonkkang@gmail.com)

**Table of Contents**

**S1 Table.** Cohort characteristics before IPTW weighting (n=3,097,179)

**S2 Table.** Association between residential NDVI and incidence of mental disorders after COVID-19

**S3 Table.** Sex-stratified associations between residential NDVI and incidence of mental disorders after COVID-19

**S4 Table.** Age-stratified association between residential NDVI and incidence of mental disorders after COVID-19

**S5 Table.** Comorbidity-stratified association between residential NDVI and incidence of mental disorders after COVID-19

**S6 Table.** Region-stratified association between residential NDVI and incidence of mental disorders after COVID-19

**S1 Table.** Cohort characteristics before IPTW weighting (n=3,097,179)

| **Baseline characteristic** | **T1** | **T2** | **SMD^a^** | **T3** | **SMD^a^** |
| --- | --- | --- | --- | --- | --- |
|  |  |  | **(T2 *vs.* T1)** |  | **(T3 *vs.* T1)** |
| Total | 784,449 | 1,849,208 |  | 463,522 |  |
| Age (y), mean ± SD | 50.14±13.23 | 51.09±13.09 | 0.072 | 53.51±13.04 | 0.257 |
| Sex, n (%) |  |  | 0.003 |  | 0.010 |
| Men | 482,598 (61.52) | 1,134,923 (61.37) |  | 282,918 (61.04) |  |
| Women | 301,851 (38.48) | 714,285 (38.63) |  | 180,604 (38.96) |  |
| Region of residence, n (%) |  |  | 0.629 |  | 1.600 |
| Urban | 562,458 (71.70) | 776,270 (41.98) |  | 47,500 (10.25) |  |
| Rural | 221,991 (28.30) | 1,072,938 (58.02) |  | 416,022 (89.75) |  |
| Baseline income, n (%) |  |  | 0.067 |  | 0.118 |
| Q1 | 204,681 (26.09) | 517,778 (28.00) |  | 139,906 (30.18) |  |
| Q2 | 314,635 (40.11) | 761,466 (41.18) |  | 191,256 (41.26) |  |
| Q3 | 265,133 (33.80) | 569,964 (30.82) |  | 132,360 (28.56) |  |
| BMI, n (%) |  |  | 0.024 |  | 0.066 |
| Underweight | 19,158 (2.44) | 39,900 (2.16) |  | 8,547 (1.84) |  |
| Normal | 241,679 (30.81) | 548,275 (29.65) |  | 129,580 (27.96) |  |
| Overweight | 189,933 (24.21) | 448,994 (24.28) |  | 114,017 (24.60) |  |
| Obesity | 333,679 (42.54) | 812,039 (43.91) |  | 211,378 (45.60) |  |
| Charlson comorbidity index, n (%) |  |  | 0.048 |  | 0.141 |
| 0 | 730,925 (93.18) | 1,694,853 (91.65) |  | 412,308 (88.95) |  |
| 1 | 28,802 (3.67) | 86,014 (4.65) |  | 29,327 (6.33) |  |
| ≥2 | 24,722 (3.15) | 68,341 (3.70) |  | 21,887 (4.72) |  |
| Blood pressure, n (%) |  |  | 0.013 |  | 0.023 |
| SBP<140 and DBP<90 | 678,397 (86.48) | 1,590,857 (86.03) |  | 397,198 (85.69) |  |
| SBP≥140 or DBP≥90 | 106,052 (13.52) | 258,351 (13.97) |  | 66,324 (14.31) |  |
| Fasting blood sugar, n (%) |  |  | 0.040 |  | 0.078 |
| <100 mg/dL | 464,081 (59.16) | 1,057,702 (57.20) |  | 256,412 (55.32) |  |
| ≥100 mg/dL | 320,368 (40.84) | 791,506 (42.80) |  | 207,110 (44.68) |  |
| Glomerular filtration rate, n (%) |  |  | 0.014 |  | 0.048 |
| <60mL/min/1.73 m² | 39,848 (5.08) | 99,540 (5.38) |  | 28,623 (6.18) |  |
| ≥60mL/min/1.73 m² | 744,601 (94.92) | 1,749,668 (94.62) |  | 434,899 (93.82) |  |
| Smoking status, n (%) |  |  | <0.001 |  | <0.001 |
| Never | 777,232 (99.08) | 1,832,944 (99.12) |  | 459,591 (99.15) |  |
| Former/Current | 6,952 (0.89) | 15,449 (0.84) |  | 3,695 (0.80) |  |
| Unknown | 265 (0.03) | 815 (0.04) |  | 236 (0.05) |  |
| Alcohol consumption, n (%) |  |  | 0.046 |  | 0.093 |
| <1 day/week | 466,111 (59.42) | 1,135,909 (61.43) |  | 293,424 (63.30) |  |
| 1-2 day/week | 225,496 (28.75) | 503,172 (27.21) |  | 116,558 (25.15) |  |
| 3-4 day/week | 72,464 (9.24) | 161,940 (8.76) |  | 39,874 (8.60) |  |
| ≥5 day/week | 20,122 (2.57) | 47,367 (2.56) |  | 13,373 (2.89) |  |
| Unknown | 3 (0.03) | 3 (0.04) |  | 3 (0.06) |  |
| Physical activity, n (%) |  |  | <0.001 |  | 0.040 |
| Insufficient | 356,205 (45.41) | 836,646 (45.24) |  | 200,889 (43.34) |  |
| Sufficient | 428,006 (54.56) | 1,011,741 (54.71) |  | 262,384 (56.61) |  |
| Unknown | 238 (0.03) | 821 (0.04) |  | 249 (0.05) |  |
| Medication for hypertension, n (%) |  |  | 0.035 |  | 0.103 |
| No | 633,013 (80.70) | 1,466,044 (79.28) |  | 354,467 (76.47) |  |
| Yes | 151,436 (19.30) | 383,164 (20.72) |  | 109,055 (23.53) |  |
| Medication for diabetes mellitus, n (%) |  |  | 0.032 |  | 0.086 |
| No | 724,896 (92.41) | 1,692,733 (91.54) |  | 417,094 (89.98) |  |
| Yes | 59,553 (7.59) | 156,475 (8.46) |  | 46,428 (10.02) |  |
| Medication for dyslipidemia, n (%) |  |  | 0.004 |  | 0.010 |
| No | 702,369 (89.54) | 1,653,734 (89.43) |  | 413,602 (89.23) |  |
| Yes | 82,080 (10.46) | 195,474 (10.57) |  | 49,920 (10.77) |  |

Abbreviations: BMI, body mass index; SD, standard deviation; SMD, Standardized mean difference; T1, low NDVI group; T2, medium NDVI group; T3, high NDVI group.

^a^ SMD < 0.1 indicates no major imbalance.

**S2 Table.** Association between residential NDVI and incidence of mental disorders after COVID-19

|  |  |  |  |  |  | **HR (95% CI)** | | | |
| --- | --- | --- | --- | --- | --- | --- | --- | --- | --- |
|  | **Total** | **Event** | **Person-years** | **Incidence rate^a^** | **P for trend** | **Model 1^b^** | **P value** | **Model 2^c^** | **P value** |
| **Common mental disorders** |  |  |  |  | 0.861 |  |  |  |  |
| Low NDVI | 690,537 | 22,687 | 1,015,752 | 22.34 |  | 1.00 (reference) |  | 1.00 (reference) |  |
| Moderate NDVI | 1,619,040 | 49,869 | 2,340,797 | 21.30 |  | **0.97 (0.96-0.98)** | <0.001 | **0.97 (0.96-0.98)** | <0.001 |
| High NDVI | 401,736 | 12,810 | 575,452 | 22.26 |  | 1.00 (0.99-1.01) | 0.640 | 1.00 (0.99-1.01) | 0.819 |
| **Anxiety disorder** |  |  |  |  | <0.001 |  |  |  |  |
| Low NDVI | 733,515 | 13,729 | 1,085,124 | 12.65 |  | 1.00 (reference) |  | 1.00 (reference) |  |
| Moderate NDVI | 1,721,499 | 30,682 | 2,501,033 | 12.27 |  | **0.98 (0.97-0.99)** | <0.001 | **0.98 (0.96-0.99)** | <0.001 |
| High NDVI | 427,876 | 8,401 | 615,689 | 13.65 |  | **1.05 (1.03-1.06)** | <0.001 | **1.04 (1.03-1.06)** | <0.001 |
| **Insomnia** |  |  |  |  | <0.001 |  |  |  |  |
| Low NDVI | 755,459 | 8,046 | 1,121,630 | 7.17 |  | 1.00 (reference) |  | 1.00 (reference) |  |
| Moderate NDVI | 1,776,317 | 18,447 | 2,589,361 | 7.12 |  | **0.97 (0.95-0.98)** | <0.001 | **0.97 (0.95-0.98)** | <0.001 |
| High NDVI | 444,043 | 4,827 | 641,524 | 7.52 |  | **0.96 (0.95-0.98)** | <0.001 | **0.97 (0.95-0.98)** | <0.001 |
| **Bipolar disorder** |  |  |  |  | <0.001 |  |  |  |  |
| Low NDVI | 780,950 | 817 | 1,163,722 | 0.70 |  | 1.00 (reference) |  | 1.00 (reference) |  |
| Moderate NDVI | 1,841,826 | 1,502 | 2,694,216 | 0.56 |  | **0.84 (0.79-0.88)** | <0.001 | **0.84 (0.79-0.88)** | <0.001 |
| High NDVI | 461,588 | 364 | 669,560 | 0.54 |  | **0.77 (0.73-0.81)** | <0.001 | **0.78 (0.74-0.82)** | <0.001 |
| **Depression** |  |  |  |  | 0.101 |  |  |  |  |
| Low NDVI | 750,060 | 9,519 | 1,112,488 | 8.56 |  | 1.00 (reference) |  | 1.00 (reference) |  |
| Moderate NDVI | 1,768,048 | 20,207 | 2,576,104 | 7.84 |  | **0.97 (0.96-0.98)** | <0.001 | **0.97 (0.96-0.99)** | <0.001 |
| High NDVI | 442,898 | 4,807 | 639,972 | 7.51 |  | 0.99 (0.97-1.00) | 0.068 | 0.99 (0.97-1.00) | 0.107 |

Abbreviations: COVID-19, coronavirus disease 2019; CI, confidence interval; HRs, hazard ratios; NDVI, normalized difference vegetation index

**^a^** Incidence rate is expressed as per 1,000 person-years.

**^b^**Adjusted HR: adjusted for age, and sex.

**^c^**Adjusted HR: adjusted for age, sex, region of residence, household income, body mass index, blood pressure, fasting blood glucose levels, estimated glomerular filtration rate, Charlson comorbidity index, history of antihypertensive medications/diabetes/dyslipidemia, smoking status, weekly alcohol consumption frequency, and aerobic physical activity level.

Bolded values indicate statistically significant differences (p-value < 0.05)

**S3 Table.** Sex-stratified associations between residential NDVI and incidence of mental disorders after COVID-19

|  |  |  |  |  |  | **HR (95% CI)** | | | |
| --- | --- | --- | --- | --- | --- | --- | --- | --- | --- |
|  | **Total** | **Event** | **Person-years** | **Incidence rate^a^** | **P for trend** | **Model 1^b^** | **P value** | **Model 2^c^** | **P value** |
| **Common mental disorders** |  |  |  |  |  |  | 0.581 |  | 0.846 |
| Male |  |  |  |  | 0.157 |  |  |  |  |
| Low NDVI | 436,760 | 11,754 | 646,118 | 18.19 |  | 1.00 (reference) |  | 1.00 (reference) |  |
| Moderate NDVI | 1,023,129 | 25,855 | 1,484,176 | 17.42 |  | **0.98 (0.96-0.99)** | <0.001 | **0.98 (0.96-0.99)** | <0.001 |
| High NDVI | 252,774 | 6,617 | 362,437 | 18.26 |  | 0.99 (0.98-1.01) | 0.208 | 0.99 (0.98-1.00) | 0.160 |
| Female |  |  |  |  | 0.063 |  |  |  |  |
| Low NDVI | 253,777 | 10,933 | 369,634 | 29.58 |  | 1.00 (reference) |  | 1.00 (reference) |  |
| Moderate NDVI | 595,911 | 24,014 | 856,621 | 28.03 |  | **0.96 (0.95-0.97)** | <0.001 | **0.96 (0.95-0.97)** | <0.001 |
| High NDVI | 148,962 | 6,193 | 213,015 | 29.07 |  | **1.02 (1.00-1.03)** | **0.028** | 1.01 (1.00-1.03) | 0.052 |
| **Anxiety disorder** |  |  |  |  |  |  | 0.723 |  | 0.700 |
| Male |  |  |  |  | <0.001 |  |  |  |  |
| Low NDVI | 457,162 | 6,914 | 679,496 | 10.18 |  | 1.00 (reference) |  | 1.00 (reference) |  |
| Moderate NDVI | 1,071,676 | 15,481 | 1,560,919 | 9.92 |  | 0.99 (0.97-1.01) | 0.281 | 0.99 (0.97-1.01) | 0.222 |
| High NDVI | 265,483 | 4,179 | 382,059 | 10.94 |  | **1.06 (1.04-1.08)** | <0.001 | **1.06 (1.04-1.08)** | <0.001 |
| Female |  |  |  |  | 0.002 |  |  |  |  |
| Low NDVI | 276,353 | 6,815 | 405,629 | 16.80 |  | 1.00 (reference) |  | 1.00 (reference) |  |
| Moderate NDVI | 649,823 | 15,201 | 940,114 | 16.17 |  | **0.97 (0.95-0.98)** | <0.001 | **0.96 (0.95-0.98)** | <0.001 |
| High NDVI | 162,393 | 4,222 | 233,630 | 18.07 |  | **1.04 (1.02-1.05)** | <0.001 | **1.03 (1.01-1.05)** | 0.002 |
| **Insomnia** |  |  |  |  |  |  | 0.009 |  | 0.039 |
| Male |  |  |  |  | 0.043 |  |  |  |  |
| Low NDVI | 468,999 | 4,038 | 699,188 | 5.78 |  | 1.00 (reference) |  | 1.00 (reference) |  |
| Moderate NDVI | 1,100,661 | 9,323 | 1,607,414 | 5.80 |  | 1.00 (0.98-1.02) | <0.001 | **0.94 (0.92-0.96)** | <0.001 |
| High NDVI | 273,287 | 2,510 | 394,435 | 6.36 |  | **0.98 (0.95-1.00)** | <0.001 | **0.95 (0.93-0.98)** | <0.001 |
| Female |  |  |  |  | <0.001 |  |  |  |  |
| Low NDVI | 286,460 | 4,008 | 422,442 | 9.49 |  | 1.00 (reference) |  | 1.00 (reference) |  |
| Moderate NDVI | 675,656 | 9,124 | 981,947 | 9.29 |  | **0.94 (0.92-0.96)** | <0.001 | **0.94 (0.92-0.96)** | <0.001 |
| High NDVI | 170,756 | 2,317 | 247,090 | 9.38 |  | **0.95 (0.93-0.98)** | <0.001 | **0.96 (0.94-0.98)** | <0.001 |
| **Bipolar disorder** |  |  |  |  |  |  | 0.106 |  | 0.126 |
| Male |  |  |  |  | <0.001 |  |  |  |  |
| Low NDVI | 480,973 | 412 | 719,224 | 0.57 |  | 1.00 (reference) |  | 1.00 (reference) |  |
| Moderate NDVI | 1,131,467 | 733 | 1,657,143 | 0.44 |  | **0.79 (0.73-0.85)** | <0.001 | **0.79 (0.74-0.85)** | <0.001 |
| High NDVI | 281,923 | 198 | 408,241 | 0.49 |  | **0.83 (0.77-0.89)** | <0.001 | **0.84 (0.78-0.91)** | <0.001 |
| Female |  |  |  |  | <0.001 |  |  |  |  |
| Low NDVI | 299,977 | 405 | 444,498 | 0.91 |  | 1.00 (reference) |  | 1.00 (reference) |  |
| Moderate NDVI | 710,359 | 769 | 1,037,072 | 0.74 |  | **0.89 (0.83-0.96)** | 0.003 | **0.89 (0.83-0.96)** | 0.002 |
| High NDVI | 179,665 | 166 | 261,319 | 0.64 |  | **0.71 (0.66-0.77)** | <0.001 | **0.71 (0.66-0.77)** | <0.001 |
| **Depression** |  |  |  |  |  |  | 0.241 |  | 0.213 |
| Male |  |  |  |  | <0.001 |  |  |  |  |
| Low NDVI | 467,237 | 4,631 | 696,082 | 6.65 |  | 1.00 (reference) |  | 1.00 (reference) |  |
| Moderate NDVI | 1,099,463 | 9,610 | 1,605,406 | 5.99 |  | **0.95 (0.93-0.97)** | <0.001 | **0.95 (0.93-0.97)** | <0.001 |
| High NDVI | 273,935 | 2,296 | 395,579 | 5.80 |  | **0.96 (0.94-0.98)** | <0.001 | **0.96 (0.94-0.98)** | <0.001 |
| Female |  |  |  |  | 0.128 |  |  |  |  |
| Low NDVI | 282,823 | 4,888 | 416,406 | 11.74 |  | 1.00 (reference) |  | 1.00 (reference) |  |
| Moderate NDVI | 668,585 | 10,597 | 970,698 | 10.92 |  | 0.99 (0.97-1.01) | 0.403 | 0.99 (0.97-1.01) | 0.421 |
| High NDVI | 168,963 | 2,511 | 244,393 | 10.27 |  | 1.02 (0.99-1.04) | 0.169 | 1.02 (1.00-1.04) | 0.123 |

Abbreviations: COVID-19, coronavirus disease 2019; CI, confidence interval; HRs, hazard ratios; NDVI, normalized difference vegetation index

**^a^** Incidence rate is expressed as per 1,000 person-years.

**^b^**Adjusted HR: adjusted for age.

**^c^**Adjusted HR: adjusted for age, region of residence, household income, body mass index, blood pressure, fasting blood glucose levels, estimated glomerular filtration rate, Charlson comorbidity index, history of antihypertensive medications/diabetes/dyslipidemia, smoking status, weekly alcohol consumption frequency, and aerobic physical activity level.

Bolded values indicate statistically significant differences (p-value < 0.05)

**S4 Table.** Age-stratified association between residential NDVI and incidence of mental disorders after COVID-19

|  |  |  |  |  |  | **HR (95% CI)** | | | |
| --- | --- | --- | --- | --- | --- | --- | --- | --- | --- |
|  | **Total** | **Event** | **Person-years** | **Incidence rate^a^** | **P for trend** | **Model 1^b^** | **P value** | **Model 2^c^** | **P value** |
| **Common mental disorders** |  |  |  |  |  |  | <0.001 |  | <0.001 |
| 19-39 years |  |  |  |  | <0.001 |  |  |  |  |
| Low NDVI | 171,117 | 5,894 | 254,907 | 23.12 |  | 1.00 (reference) |  | 1.00 (reference) |  |
| Moderate NDVI | 352,332 | 10,702 | 520,149 | 20.58 |  | **0.93 (0.91-0.94)** | <0.001 | **0.93 (0.91-0.94)** | <0.001 |
| High NDVI | 68,304 | 2,061 | 100,465 | 20.52 |  | **0.92 (0.90-0.94)** | <0.001 | **0.92 (0.90-0.94)** | <0.001 |
| 40-59 years |  |  |  |  | 0.074 |  |  |  |  |
| Low NDVI | 329,431 | 9,836 | 489,581 | 20.09 |  | 1.00 (reference) |  | 1.00 (reference) |  |
| Moderate NDVI | 795,522 | 22,321 | 1,164,701 | 19.17 |  | 0.96 (0.95-0.98) | <0.001 | 0.96 (0.95-0.98) | <0.001 |
| High NDVI | 186,723 | 5,358 | 271,847 | 19.71 |  | 1.01 (1.00-1.03) | 0.085 | 1.01 (1.00-1.03) | 0.070 |
| ≥60 years |  |  |  |  | <0.001 |  |  |  |  |
| Low NDVI | 189,989 | 6,957 | 271,264 | 25.65 |  | 1.00 (reference) |  | 1.00 (reference) |  |
| Moderate NDVI | 471,186 | 16,846 | 655,947 | 25.68 |  | 1.01 (1.00-1.03) | 0.182 | 1.01 (0.99-1.03) | 0.263 |
| High NDVI | 146,709 | 5,391 | 203,140 | 26.54 |  | **1.05 (1.03-1.07)** | <0.001 | **1.05 (1.03-1.06)** | <0.001 |
| **Anxiety disorder** |  |  |  |  |  |  | <0.001 |  | 0.002 |
| 19-39 years |  |  |  |  | 0.688 |  |  |  |  |
| Low NDVI | 179,737 | 3,565 | 269,437 | 13.23 |  | 1.00 (reference) |  | 1.00 (reference) |  |
| Moderate NDVI | 367,654 | 6,593 | 545,665 | 12.08 |  | **0.96 (0.93-0.98)** | 0.001 | **0.96 (0.94-0.98)** | 0.002 |
| High NDVI | 71,038 | 1,362 | 104,938 | 12.98 |  | 0.99 (0.97-1.02) | 0.569 | 1.00 (0.97-1.02) | 0.679 |
| 40-59 years |  |  |  |  | <0.001 |  |  |  |  |
| Low NDVI | 346,202 | 6,049 | 517,369 | 11.69 |  | 1.00 (reference) |  | 1.00 (reference) |  |
| Moderate NDVI | 836,785 | 13,932 | 1,231,104 | 11.32 |  | **0.97 (0.96-0.99)** | 0.003 | **0.97 (0.96-0.99)** | 0.002 |
| High NDVI | 196,174 | 3,499 | 286,973 | 12.19 |  | **1.04 (1.02-1.06)** | <0.001 | **1.04 (1.02-1.06)** | <0.001 |
| ≥60 years |  |  |  |  | <0.001 |  |  |  |  |
| Low NDVI | 207,576 | 4,115 | 298,319 | 13.79 |  | 1.00 (reference) |  | 1.00 (reference) |  |
| Moderate NDVI | 517,060 | 10,157 | 724,264 | 14.02 |  | 1.00 (0.98-1.02) | 0.873 | 1.00 (0.98-1.02) | 0.778 |
| High NDVI | 160,664 | 3,540 | 223,778 | 15.82 |  | **1.09 (1.07-1.12)** | <0.001 | **1.08 (1.06-1.10)** | <0.001 |
| **Insomnia** |  |  |  |  |  |  | <0.001 |  | 0.001 |
| 19-39 years |  |  |  |  | <0.001 |  |  |  |  |
| Low NDVI | 187,219 | 1,027 | 282,372 | 3.64 |  | 1.00 (reference) |  | 1.00 (reference) |  |
| Moderate NDVI | 382,670 | 1,751 | 571,147 | 3.07 |  | **0.86 (0.82-0.91)** | <0.001 | **0.86 (0.82-0.91)** | <0.001 |
| High NDVI | 74,011 | 313 | 110,069 | 2.84 |  | **0.77 (0.73-0.81)** | <0.001 | **0.77 (0.74-0.81)** | <0.001 |
| 40-59 years |  |  |  |  | 0.100 |  |  |  |  |
| Low NDVI | 357,231 | 3,183 | 535,836 | 5.94 |  | 1.00 (reference) |  | 1.00 (reference) |  |
| Moderate NDVI | 863,817 | 7,609 | 1,275,362 | 5.97 |  | 0.99 (0.97-1.01) | 0.410 | 0.99 (0.97-1.02) | 0.460 |
| High NDVI | 203,123 | 1,799 | 298,253 | 6.03 |  | 1.02 (0.99-1.04) | 0.178 | 1.02 (1.00-1.05) | 0.098 |
| ≥60 years |  |  |  |  | <0.001 |  |  |  |  |
| Low NDVI | 211,009 | 3,836 | 303,423 | 12.64 |  | 1.00 (reference) |  | 1.00 (reference) |  |
| Moderate NDVI | 529,830 | 9,087 | 742,852 | 12.23 |  | **0.98 (0.95-1.00)** | 0.026 | **0.97 (0.95-1.00)** | 0.018 |
| High NDVI | 166,909 | 2,715 | 233,202 | 11.64 |  | **0.96 (0.94-0.98)** | 0.001 | **0.96 (0.94-0.98)** | <0.001 |
| **Bipolar disorder** |  |  |  |  |  |  | 0.049 |  | 0.082 |
| 19-39 years |  |  |  |  | <0.001 |  |  |  |  |
| Low NDVI | 189,266 | 323 | 285,999 | 1.13 |  | 1.00 (reference) |  | 1.00 (reference) |  |
| Moderate NDVI | 386,689 | 476 | 578,065 | 0.82 |  | **0.82 (0.75-0.90)** | <0.001 | **0.81 (0.74-0.89)** | <0.001 |
| High NDVI | 74,836 | 80 | 111,472 | 0.72 |  | **0.69 (0.62-0.75)** | <0.001 | **0.68 (0.61-0.75)** | <0.001 |
| 40-59 years |  |  |  |  | 0.004 |  |  |  |  |
| Low NDVI | 366,648 | 308 | 551,839 | 0.56 |  | 1.00 (reference) |  | 1.00 (reference) |  |
| Moderate NDVI | 887,954 | 585 | 1,315,650 | 0.45 |  | **0.81 (0.74-0.88)** | <0.001 | **0.81 (0.74-0.88)** | <0.001 |
| High NDVI | 208,869 | 150 | 307,852 | 0.49 |  | **0.88 (0.81-0.96)** | 0.003 | **0.89 (0.82-0.97)** | 0.005 |
| ≥60 years |  |  |  |  | 0.090 |  |  |  |  |
| Low NDVI | 225,036 | 186 | 325,884 | 0.57 |  | 1.00 (reference) |  | 1.00 (reference) |  |
| Moderate NDVI | 567,183 | 441 | 800,501 | 0.55 |  | 0.93 (0.84-1.02) | 0.132 | 0.93 (0.84-1.03) | 0.150 |
| High NDVI | 177,883 | 134 | 250,236 | 0.54 |  | **0.75 (0.67-0.83)** | <0.001 | **0.76 (0.68-0.84)** | <0.001 |
| **Depression** |  |  |  |  |  |  | <0.001 |  | <0.001 |
| 19-39 years |  |  |  |  | <0.001 |  |  |  |  |
| Low NDVI | 181,075 | 3,243 | 271,761 | 11.93 |  | 1.00 (reference) |  | 1.00 (reference) |  |
| Moderate NDVI | 372,286 | 5,575 | 553,579 | 10.07 |  | **0.89 (0.87-0.92)** | <0.001 | **0.89 (0.87-0.92)** | <0.001 |
| High NDVI | 72,348 | 993 | 107,238 | 9.26 |  | **0.82 (0.80-0.85)** | <0.001 | **0.82 (0.80-0.85)** | <0.001 |
| 40-59 years |  |  |  |  | 0.090 |  |  |  |  |
| Low NDVI | 354,490 | 3,978 | 531,205 | 7.49 |  | 1.00 (reference) |  | 1.00 (reference) |  |
| Moderate NDVI | 858,617 | 8,716 | 1,267,100 | 6.88 |  | **0.96 (0.94-0.99)** | 0.002 | **0.96 (0.94-0.99)** | 0.001 |
| High NDVI | 202,528 | 1,954 | 297,366 | 6.57 |  | 1.02 (1.00-1.04) | 0.119 | 1.02 (1.00-1.04) | 0.087 |
| ≥60 years |  |  |  |  | <0.001 |  |  |  |  |
| Low NDVI | 214,495 | 2,298 | 309,522 | 7.42 |  | 1.00 (reference) |  | 1.00 (reference) |  |
| Moderate NDVI | 537,145 | 5,916 | 755,425 | 7.83 |  | **1.08 (1.05-1.11)** | <0.001 | **1.08 (1.05-1.11)** | <0.001 |
| High NDVI | 168,022 | 1,860 | 235,369 | 7.90 |  | **1.14 (1.11-1.17)** | <0.001 | **1.15 (1.12-1.18)** | <0.001 |

Abbreviations: COVID-19, coronavirus disease 2019; CI, confidence interval; HRs, hazard ratios; NDVI, normalized difference vegetation index

**^a^** Incidence rate is expressed as per 1,000 person-years.

**^b^**Adjusted HR: adjusted for sex.

**^c^**Adjusted HR: adjusted for sex, region of residence, household income, body mass index, blood pressure, fasting blood glucose levels, estimated glomerular filtration rate, Charlson comorbidity index, history of antihypertensive medications/diabetes/dyslipidemia, smoking status, weekly alcohol consumption frequency, and aerobic physical activity level.

Bolded values indicate statistically significant differences (p-value < 0.05)

**S5 Table.** Comorbidity-stratified association between residential NDVI and incidence of mental disorders after COVID-19

|  |  |  |  |  |  | **HR (95% CI)** | | | |
| --- | --- | --- | --- | --- | --- | --- | --- | --- | --- |
|  | **Total** | **Event** | **Person-years** | **Incidence rate^a^** | **P for trend** | **Model 1^b^** | **P value** | **Model 2^c^** | **P value** |
| **Common mental disorders** |  |  |  |  |  |  | 0.005 |  | 0.014 |
| Without comorbidities |  |  |  |  | 0.143 |  |  |  |  |
| Low NDVI | 646,848 | 20,940 | 954,850 | 21.93 |  | 1.00 (reference) |  | 1.00 (reference) |  |
| Moderate NDVI | 1,494,951 | 45,242 | 2,171,774 | 20.83 |  | **0.97 (0.96-0.98)** | <0.001 | **0.97 (0.96-0.98)** | <0.001 |
| High NDVI | 361,107 | 11,136 | 520,662 | 21.39 |  | 0.99 (0.98-1.00) | 0.127 | 0.99 (0.98-1.00) | 0.160 |
| With comorbidities |  |  |  |  | <0.001 |  |  |  |  |
| Low NDVI | 43,689 | 1,747 | 60,902 | 26.69 |  | 1.00 (reference) |  | 1.00 (reference) |  |
| Moderate NDVI | 124,089 | 4,627 | 169,023 | 27.38 |  | **0.97 (0.96-0.98)** | <0.001 | **0.97 (0.96-0.98)** | <0.001 |
| High NDVI | 40,629 | 1,674 | 54,790 | 30.55 |  | **1.09 (1.06-1.12)** | <0.001 | **1.09 (1.06-1.14)** | <0.001 |
| **Anxiety disorder** |  |  |  |  |  |  | 0.256 |  | 0.301 |
| Without comorbidities |  |  |  |  | <0.001 |  |  |  |  |
| Low NDVI | 20,394 | 745 | 27,562 | 27.03 |  | 1.00 (reference) |  | 1.00 (reference) |  |
| Moderate NDVI | 55,574 | 2,014 | 73,274 | 27.49 |  | 1.04 (0.99-1.09) | 0.115 | 1.04 (0.99-1.09) | 0.151 |
| High NDVI | 17,415 | 672 | 22,772 | 29.51 |  | **1.12 (1.07-1.17)** | <0.001 | **1.12 (1.07-1.17)** | <0.001 |
| With comorbidities |  |  |  |  | 0.001 |  |  |  |  |
| Low NDVI | 48,331 | 1,065 | 67,820 | 15.76 |  | 1.00 (reference) |  | 1.00 (reference) |  |
| Moderate NDVI | 138,097 | 2,938 | 189,298 | 15.52 |  | 0.99 (0.95-1.03) | 0.655 | 0.99 (0.95-1.03) | 0.630 |
| High NDVI | 45,267 | 1,076 | 61,428 | 17.52 |  | **1.07 (1.03-1.11)** | <0.001 | **1.07 (1.03-1.10)** | 0.001 |
| **Insomnia** |  |  |  |  |  |  | 0.467 |  | 0.489 |
| Without comorbidities |  |  |  |  | <0.001 |  |  |  |  |
| Low NDVI | 25,827 | 641 | 37,216 | 17.22 |  | 1.00 (reference) |  | 1.00 (reference) |  |
| Moderate NDVI | 76,332 | 1,749 | 107,287 | 16.30 |  | **0.95 (0.90-1.00)** | 0.043 | **0.95 (0.90-1.00)** | 0.046 |
| High NDVI | 25,764 | 646 | 35,801 | 18.04 |  | 0.96 (0.91-1.01) | 0.103 | 0.95 (0.91-1.00) | 0.058 |
| With comorbidities |  |  |  |  | 0.756 |  |  |  |  |
| Low NDVI | 49,831 | 884 | 70,122 | 12.61 |  | 1.00 (reference) |  | 1.00 (reference) |  |
| Moderate NDVI | 143,175 | 2,300 | 196,834 | 11.69 |  | **0.94 (0.90-0.99)** | 0.009 | **0.95 (0.91-0.99)** | 0.012 |
| High NDVI | 47,411 | 812 | 64,666 | 12.56 |  | 0.99 (0.94-1.03) | 0.516 | 0.99 (0.95-1.04) | 0.770 |
| **Bipolar disorder** |  |  |  |  |  |  | 0.037 |  | 0.055 |
| Without comorbidities |  |  |  |  | <0.001 |  |  |  |  |
| Low NDVI | 705,628 | 7,162 | 1,051,508 | 6.81 |  | 1.00 (reference) |  | 1.00 (reference) |  |
| Moderate NDVI | 1,633,142 | 16,147 | 2,392,527 | 6.75 |  | **0.97 (0.96-0.99)** | <0.001 | **0.97 (0.96-0.99)** | 0.001 |
| High NDVI | 396,632 | 4,015 | 576,859 | 6.96 |  | **0.96 (0.94-0.98)** | <0.001 | **0.96 (0.95-0.98)** | <0.001 |
| With comorbidities |  |  |  |  | 0.430 |  |  |  |  |
| Low NDVI | 53,068 | 77 | 75,170 | 1.02 |  | 1.00 (reference) |  | 1.00 (reference) |  |
| Moderate NDVI | 153,055 | 199 | 211,625 | 0.94 |  | **0.83 (0.72-0.96)** | 0.010 | **0.83 (0.72-0.96)** | 0.010 |
| High NDVI | 50,757 | 80 | 69,630 | 1.15 |  | 0.94 (0.81-1.08) | 0.351 | 0.92 (0.82-1.09) | 0.447 |
| **Depression** |  |  |  |  |  |  | 0.137 |  | 0.241 |
| Without comorbidities |  |  |  |  | <0.001 |  |  |  |  |
| Low NDVI | 23,057 | 410 | 31,407 | 13.06 |  | 1.00 (reference) |  | 1.00 (reference) |  |
| Moderate NDVI | 63,432 | 1,040 | 84,380 | 12.33 |  | 0.98 (0.92-1.05) | 0.574 | 0.99 (0.92-1.05) | 0.667 |
| High NDVI | 20,208 | 351 | 26,704 | 13.14 |  | 0.99 (0.92-1.06) | 0.693 | 1.00 (0.93-1.07) | 0.923 |
| With comorbidities |  |  |  |  | <0.001 |  |  |  |  |
| Low NDVI | 49,860 | 713 | 90,250 | 10.15 |  | 1.00 (reference) |  | 1.00 (reference) |  |
| Moderate NDVI | 142,845 | 1,904 | 196,558 | 9.69 |  | 1.01 (0.96-1.06) | 0.826 | 1.01 (0.96-1.06) | 0.839 |
| High NDVI | 47,409 | 651 | 64,708 | 10.06 |  | **1.18 (1.12-1.24)** | <0.001 | **1.19 (1.13-1.25)** | <0.001 |

Abbreviations: COVID-19, coronavirus disease 2019; CI, confidence interval; HRs, hazard ratios; NDVI, normalized difference vegetation index

**^a^** Incidence rate is expressed as per 1,000 person-years.

**^b^**Adjusted HR: adjusted for age, and sex.

**^c^**Adjusted HR: adjusted for sex, region of residence, household income, body mass index, blood pressure, fasting blood glucose levels, estimated glomerular filtration rate, history of antihypertensive medications/diabetes/dyslipidemia, smoking status, weekly alcohol consumption frequency, and aerobic physical activity level.

Bolded values indicate statistically significant differences (p-value < 0.05)

**S6 Table.** Region-stratified association between residential NDVI and incidence of mental disorders after COVID-19

|  |  |  |  |  |  | **HR (95% CI)** | | | |
| --- | --- | --- | --- | --- | --- | --- | --- | --- | --- |
|  | **Total** | **Event** | **Person-years** | **Incidence rate^a^** | **P for trend** | **Model 1^b^** | **P value** | **Model 2^c^** | **P value** |
| **Common mental disorders** |  |  |  |  |  |  | 0.008 |  | 0.007 |
| Urban |  |  |  |  | 0.028 |  |  |  |  |
| Low NDVI | 493,888 | 16,684 | 730,638 | 22.84 |  | 1.00 (reference) |  | 1.00 (reference) |  |
| Moderate NDVI | 676,777 | 21,427 | 979,343 | 21.88 |  | **0.95 (0.94-0.96)** | <0.001 | **0.95 (0.94-0.96)** | <0.001 |
| High NDVI | 41,244 | 1,304 | 57,170 | 22.81 |  | **0.99 (0.97-1.00)** | 0.042 | **0.99 (0.97-1.00)** | 0.039 |
| Rural |  |  |  |  | 0.033 |  |  |  |  |
| Low NDVI | 196,649 | 6,003 | 285,114 | 21.06 |  | 1.00 (reference) |  | 1.00 (reference) |  |
| Moderate NDVI | 942,263 | 28,442 | 1,361,454 | 20.89 |  | **0.99 (0.97-1.00)** | <0.001 | **0.98 (0.97-1.00)** | <0.001 |
| High NDVI | 360,492 | 11,506 | 518,282 | 22.20 |  | **1.02 (1.00-1.03)** | <0.001 | **1.01 (1.00-1.03)** | <0.001 |
| **Anxiety disorder** |  |  |  |  |  |  | 0.007 |  | 0.007 |
| Urban |  |  |  |  | 0.090 |  |  |  |  |
| Low NDVI | 526,035 | 10,039 | 782,789 | 12.83 |  | 1.00 (reference) |  | 1.00 (reference) |  |
| Moderate NDVI | 720,619 | 13,298 | 1,048,006 | 12.69 |  | 0.99 (0.97-1.00) | 0.095 | 0.99 (0.97-1.00) | 0.089 |
| High NDVI | 44,048 | 776 | 61,325 | 12.65 |  | 0.99 (0.97-1.01) | 0.202 | 0.99 (0.97-1.00) | 0.093 |
| Rural |  |  |  |  | <0.001 |  |  |  |  |
| Low NDVI | 207,480 | 3,690 | 302,335 | 12.21 |  | 1.00 (reference) |  | 1.00 (reference) |  |
| Moderate NDVI | 1,000,880 | 17,384 | 1,453,027 | 11.96 |  | **0.97 (0.96-0.99)** | 0.001 | **0.97 (0.95-0.99)** | <0.001 |
| High NDVI | 383,828 | 7,625 | 554,364 | 13.75 |  | **1.09 (1.08-1.11)** | <0.001 | **1.09 (1.07-1.11)** | <0.001 |
| **Insomnia** |  |  |  |  |  |  | 0.007 |  | 0.007 |
| Urban |  |  |  |  | 0.312 |  |  |  |  |
| Low NDVI | 541,436 | 5,914 | 808,690 | 7.31 |  | 1.00 (reference) |  | 1.00 (reference) |  |
| Moderate NDVI | 744,806 | 7,824 | 1,086,988 | 7.20 |  | **0.94 (0.92-0.97)** | <0.001 | **0.95 (0.92-0.97)** | <0.001 |
| High NDVI | 45,432 | 515 | 63,432 | 8.12 |  | 1.01 (0.99-1.04) | 0.297 | 1.01 (0.99-1.04) | 0.232 |
| Rural |  |  |  |  | <0.001 |  |  |  |  |
| Low NDVI | 214,023 | 2,132 | 312,939 | 6.81 |  | 1.00 (reference) |  | 1.00 (reference) |  |
| Moderate NDVI | 1,031,511 | 10,623 | 1,502,373 | 7.07 |  | 0.99 (0.97-1.01) | 0.284 | 0.99 (0.97-1.01) | 0.333 |
| High NDVI | 398,611 | 4,312 | 578,092 | 7.46 |  | **0.93 (0.91-0.95)** | <0.001 | **0.93 (0.91-0.95)** | <0.001 |
| **Bipolar disorder** |  |  |  |  |  |  | 0.114 |  | 0.114 |
| Urban |  |  |  |  | <0.001 |  |  |  |  |
| Low NDVI | 559,852 | 626 | 839,347 | 0.75 |  | 1.00 (reference) |  | 1.00 (reference) |  |
| Moderate NDVI | 773,201 | 677 | 1,132,477 | 0.60 |  | **0.80 (0.74-0.86)** | <0.001 | **0.79 (0.73-0.85)** | <0.001 |
| High NDVI | 47,327 | 31 | 66,317 | 0.47 |  | **0.63 (0.58-0.68)** | <0.001 | **0.64 (0.59-0.69)** | <0.001 |
| Rural |  |  |  |  | <0.001 |  |  |  |  |
| Low NDVI | 221,098 | 191 | 324,375 | 0.59 |  | 1.00 (reference) |  | 1.00 (reference) |  |
| Moderate NDVI | 1,068,625 | 825 | 1,561,739 | 0.53 |  | **0.88 (0.82-0.95)** | 0.001 | **0.89 (0.82-0.96)** | 0.002 |
| High NDVI | 414,261 | 333 | 603,243 | 0.55 |  | **0.91 (0.84-0.98)** | 0.009 | **0.91 (0.85-0.98)** | 0.012 |
| **Depression** |  |  |  |  |  |  | <0.001 |  | <0.001 |
| Urban |  |  |  |  | <0.001 |  |  |  |  |
| Low NDVI | 536,364 | 7,176 | 800,185 | 8.97 |  | 1.00 (reference) |  | 1.00 (reference) |  |
| Moderate NDVI | 741,480 | 8,741 | 1,081,658 | 8.08 |  | **0.91 (0.89-0.93)** | <0.001 | **0.91 (0.89-0.93)** | <0.001 |
| High NDVI | 45,297 | 555 | 63,188 | 8.78 |  | 0.98 (0.96-1.00) | 0.063 | 0.98 (0.96-1.01) | 0.136 |
| Rural |  |  |  |  | 0.011 |  |  |  |  |
| Low NDVI | 213,696 | 2,343 | 312,303 | 7.50 |  | 1.00 (reference) |  | 1.00 (reference) |  |
| Moderate NDVI | 1,026,568 | 11,466 | 1,494,446 | 7.67 |  | **1.03 (1.01-1.06)** | 0.002 | **1.03 (1.01-1.06)** | 0.002 |
| High NDVI | 397,601 | 4,252 | 576,784 | 7.37 |  | 0.99 (0.97-1.02) | 0.552 | 0.99 (0.97-1.01) | 0.502 |

Abbreviations: COVID-19, coronavirus disease 2019; CI, confidence interval; HRs, hazard ratios; NDVI, normalized difference vegetation index

**^a^** Incidence rate is expressed as per 1,000 person-years.

**^b^**Adjusted HR: adjusted for sex and age.

**^c^**Adjusted HR: adjusted for sex, age, household income, body mass index, blood pressure, fasting blood glucose levels, estimated glomerular filtration rate, Charlson comorbidity index, history of antihypertensive medications/diabetes/dyslipidemia, smoking status, weekly alcohol consumption frequency, and aerobic physical activity level.

Bolded values indicate statistically significant differences (p-value < 0.05)
